# Systematic realist synthesis of health-related and lifestyle interventions designed to decrease overweight, obesity and unemployment in adults

**DOI:** 10.1186/s12889-022-14518-6

**Published:** 2022-11-17

**Authors:** Sophia D. Amenyah, Diane Waters, Wen Tang, Lee-Ann Fenge, Jane L. Murphy

**Affiliations:** 1 Faculty of Health and Social Sciences, Bournemouth University, 5th Floor, Bournemouth Gateway Building, St Paul’s Lane, Bournemouth, BH8 8GP UK; 2 Faculty of Science and Technology, Bournemouth University, Talbot Campus, Fern Barrow, Poole, BH12 5BB UK

**Keywords:** Realist synthesis, Obesity, Unemployment, Overweight, Health-related interventions

## Abstract

**Background:**

Obesity and unemployment are complex social and health issues with underlying causes that are interconnected. While a clear link has been established, there is lack of evidence on the underlying causal pathways and how health-related interventions could reduce obesity and unemployment using a holistic approach.

**Objectives:**

The aim of this realist synthesis was to identify the common strategies used by health-related interventions to reduce obesity, overweight and unemployment and to determine for whom and under what circumstances these interventions were successful or unsuccessful and why.

**Methods:**

A realist synthesis approach was used. Systematic literature searches were conducted in Cochrane library, Medline, SocIndex, Cumulative Index to Nursing and Allied Health Literature (CINAHL), Scopus, and PsychInfo. The evidence from included studies were synthesised into Context-Mechanism-Outcome configurations (CMOcs) to better understand when and how programmes work, for which participants and to refine the final programme theory.

**Results:**

A total of 83 articles met the inclusion criteria. 8 CMOcs elucidating the contexts of the health-related interventions, underlying mechanisms and outcomes were identified. Interventions that were tailored to the target population using multiple strategies, addressing different aspects of individual and external environments led to positive outcomes for reemployment and reduction of obesity.

**Conclusion:**

This realist synthesis presents a broad array of contexts, mechanisms underlying the success of health-related interventions to reduce obesity and unemployment. It provides novel insights and key factors that influence the success of such interventions and highlights a need for participatory and holistic approaches to maximise the effectiveness of programmes designed to reduce obesity and unemployment.

**Trial registration:**

PROSPERO 2020 CRD42020219897.

**Supplementary Information:**

The online version contains supplementary material available at 10.1186/s12889-022-14518-6.

## Background

Obesity and unemployment are critically intertwined social and health issues which adversely impact life expectancy, quality of life, mental health and lead to increased mortality and morbidity [1–4]. Whether obesity leads to unemployment or is a consequence of unemployment is not fully determined, however there is strong evidence showing that both conditions are reciprocal and can be the cause or consequence of each other [5, 6]. The recent coronavirus pandemic and cost of living crisis have exacerbated the challenges of being unemployed and living with low income [7, 8]. Furthermore, they have highlighted the risks of living with overweight and obesity and the need for interventions to address the underlying social and economic determinants [9, 10].

Several studies have shown a consistent link between obesity and unemployment [11–13] and single transitions into unemployment and persistent unemployment have been associated with poor mental health, general health and obesity [12]. In a cohort study of 87,796 participants, obesity was associated with a higher risk of unemployment and sickness absence compared with individuals with normal weight [5]. Additionally, evidence suggests that long-term obesity and developing obesity in mid-adulthood increases the risk of poor work ability [14]. Taken together, this evidence suggests that reemployment might be an important strategy to improve the health of unemployed individuals living with overweight or obesity.

Evidence on the link between obesity, income inequality and unemployment also highlight the underlying effects of obesity determinants related to dietary and physical activity behaviours. Individuals from lower socioeconomic groups are more likely to exhibit a greater risk of higher consumption of energy dense foods, lower density of micronutrients in their diet, lower consumption of fruits and vegetables and lower levels of physical activity [15–17]. Unemployment has an immediate effect on food expenditure and longitudinal data showed that this decreased with the duration of unemployment and is also associated with the purchase of cheaper, energy dense foods but lower purchase of fruits and vegetables [6, 18]. A review on neighbourhood disparities in access to fast-food outlets and convenience shops showed that, low-income neighbourhoods offered greater access to food sources that promote unhealthy eating thereby worsening the problem [19]. Compared to the general population, unemployed persons are more sedentary and show lower levels of physical activity [20, 21].

The underlying causes of obesity and unemployment are similar and often very complex. Similar to the challenge of maintaining a healthy weight, finding employment or reemployment after job loss is a complex and difficult task that requires extensive motivation and self-regulation [22, 23]. Secondly obesity and job loss impact on certain characteristics, like self-esteem and self-efficacy and this negatively influences access to employment and reduces performance in the labour market [4, 24]. Individuals living with obesity or in long-term unemployment may also be discriminated against due to prejudice and stereotyping by employers [25–27], further decreasing their chances of obtaining employment and earning an income to enable the maintenance of a healthy lifestyle. Unemployment, low income and obesity are also associated with higher levels of psychosocial stressors for example, decreased control over life, higher insecurity, social isolation, stress and mental disorders [28]. This may lead to maladaptive coping strategies, such as eating energy-dense foods to alleviate negative emotions and stress resulting in a vicious cycle of overweight and unemployment [29]. This requires a range of interventions to address the complex interplay between socioeconomic factors, disadvantage, health and wellbeing. These include interventions that address skills, availability and access to healthy food options, availability and access to physical activity resources, neighbourhood safety, stress, discrimination, and dysfunctional social networks. Holistic multicomponent responses across these domains have the potential to be benefit both obese and unemployed individuals.

Currently, research gaps exist on the mechanisms and pathways that underscore the complex relationship between food insecurity, unemployment, low income, diet, and weight outcomes. There is also a lack of synthesised evidence on how health-related interventions could reduce obesity and increase employment. While some systematic reviews [30, 31] have suggested a beneficial effect of interventions in reducing obesity and increasing employment, the evidence has been inconclusive. It is also not clear which contexts or mechanisms are required for the successful implementation and effective uptake of such interventions. There is, therefore, the need to synthesise the evidence on interventions that have been shown to reduce obesity and increase employment to examine why and how these interventions worked and for whom.

### Research questions

1. What health-related interventions have been used to reduce overweight, obesity and unemployment in adults?
2. What are the common approaches used in interventions designed to reduce overweight, obesity and unemployment in adults?
3. What are the contexts and mechanisms that have contributed to the success or failure of these interventions?

### Study objectives

The objectives of this realist systematic review were to synthesise the current evidence on health-related interventions designed to reduce obesity and unemployment. Additionally, this study explored the contexts and mechanisms which underly the effectiveness of such interventions and summarised the common strategies that have been used to address obesity and unemployment.

## Methods

This realist synthesis was conducted using steps outlined in the Ray Pawson’s realist review method [32] and according to the Realist And MEta-narrative Evidence Syntheses: Evolving Standards (RAMESES) quality standards for realist synthesis [33] and a registered protocol published in the Prospective Register of Systematic Reviews (PROSPERO; CRD42020219897). Reporting was carried out using the RAMESES publication standards [34] (Supplementary Table S1) and the Preferred Reporting Items for Systematic Reviews and Meta-Analyses (PRISMA) guidelines [35] (Supplementary Table S2).

### Rationale for using realist synthesis

In order to achieve the objectives of the present review, a realist synthesis approach was chosen. Simply knowing that interventions designed to reduce obesity or unemployment work is not enough for policymakers to decide on the types of interventions to be implemented under different contexts. It is, therefore, very important to examine these interventions closely to determine which aspects led to success or failure in different circumstances and for which participants. While the majority of investigations so far may deem an intervention to work, without considering the background contexts or mechanisms in determining outcomes, such programmes may show differential results when implemented in different contexts during scaling-up. Additionally, while several systematic reviews [30, 36–39] have attempted to summarise evidence on interventions designed to reduce obesity and unemployment, the results have been inconclusive with several recommending further studies to clarify mechanisms and outcomes. This is because of unsystematic reporting within published intervention studies and the pooling of average intervention effect sizes within systematic literature reviews of studies with significant between-study heterogeneity. This results in a failure to identify effective intervention components that are specific enough and pragmatically relevant for the intervention to be scaled up where necessary [40].

In contrast, realist synthesis uses the Context-Mechanism-Outcome (CMO) heuristic in which context is the backdrop or background environment of intervention programmes [32, 41]. Mechanisms are defined as the resources generated from programme strategies and how people respond to resources offered through those strategies [32, 42]. As such, the realist approach is highly suited to clarifying what intervention approaches work, for whom, under what circumstances, and how [32]. Realist synthesis additionally lends itself to the review of complex interventions such as those designed to reduce obesity and unemployment because it accounts for context, mechanisms underlying such interventions as well as outcomes in the process of systematically and transparently synthesising relevant literature [43].

### Development of the initial programme theory

Scoping of existing literature was conducted to develop the initial programme theory (IPT) and to guide the synthesis. This involved a combination of discussions with team members with expert knowledge in the subject area, exploratory search and brief review of key articles identified at the beginning stage of the review. Initial drafts of the IPT and research questions were further discussed with project partners to further refine the aim of the proposed review according to the priorities of the partner organisations.

### Study search, screening and study selection

Screening of eligible studies, full-text assessment, data extraction, and quality appraisal of studies was independently carried out by two authors (SDA, DW). Discrepancies were discussed and resolved by consensus and, where necessary, moderated by a third reviewer from the team. Systematic searches were conducted in 6 databases including the Cochrane library, Medline, SocIndex, Cumulative Index to Nursing and Allied Health Literature (CINAHL), Scopus, and PsychInfo without any language restrictions in July 2020. These databases were included because they had been identified in the preliminary search as containing the journals relevant to the research topic. The literature search was carried out with assistance of an experienced librarian. The search was iterative and continued throughout the review. Medical subject headings and key word searches were conducted in Medline, CINAHL, SocIndex, PsychINFO and Cochrane library, whereas searches in Scopus were carried out using only key word searches. The full search strategy for all the searches combined terms related to obesity or overweight or synonyms (e.g., weight gain, weight loss, body mass index weight, body weight maintenance), unemployment or jobseeker of synonyms (e.g., unemployed, job loss, jobless) and intervention strategies (e.g., weight reduction programme, lifestyle intervention, health promotion, healthy diet, physical activity). The full searches for all the databases are provided in Supplementary Table S3. Medical subject headings and key word searches were conducted in Medline, CINAHL, SocIndex, PsychINFO and Cochrane library, whereas searches in Scopus were carried out using only key word searches.

Initial screening of titles and abstracts of the retrieved searches were conducted separately for each database and articles identified to be relevant were exported into Endnote Web for removal of duplicates. After removal of duplicates, further screening of abstracts was carried out to identify articles which were potentially relevant for inclusion in the review. Full-text articles were independently reviewed by two authors for inclusion using predefined eligibility criteria which included questions to assess a study’s relevance for inclusion in the review. Studies that described health-related or behavioural interventions (educational, skills training, health promotion, psychological, behavioural therapy, counselling) focused on promoting healthy lifestyle, wellbeing and employment in individuals were included. Full-text articles that met the inclusion criteria were added to a database for subsequent data extraction.

### Eligibility criteria

#### Inclusion criteria

- Studies conducted in adults 18 years and above living with overweight or obesity.
- Studies conducted in adults 18 years and above who are unemployed or jobseekers.

#### Exclusion criteria

- Studies involving children and adolescents below 18 years.
- Studies specifically conducted in older adults (65 years and above).
- Interventions conducted in individuals with specific health conditions.
- In-vitro or non-human studies.
- Interventions involving drugs or surgery e.g., bariatric surgery, interventions targeted at changing the food environment or fiscal and regulatory policies.

### Data extraction

Data extraction was carried out independently by two members of the team. The first stage included extracting data on study characteristics including first author, country, target group, study design, sample size, description of intervention, duration, programme theory, evaluation methods, and study outcomes. The second stage involved extracting data on contexts, mechanisms, information on the effectiveness of the interventions and facilitators and barriers for the implementation of the interventions which contributed to the refinement of the final programme theory.

### Quality appraisal

Consistent with realist synthesis methodology, quality appraisal of included studies was conducted to assess their relevance and rigour. Methodological rigour refers to whether the methods used to generate the relevant data were credible, plausible and trustworthy and relevance refers to relevance of the contributions of any section of the study to refining the underlying theory and context-mechanism-outcome evidence [32, 44]. Relevance in this synthesis was assessed by considering whether the paper had a direct relevance to our review by contributing to the final program theory. Assessment for rigour was based on the extent to which studies provided a detailed description of methods and the level of generalisability [45] of findings. Two reviewers initially appraised two articles together and discussed the results as a team to ensure a consistent approach for this process.

### Data synthesis and analysis

Data synthesis and analysis was conducted using in-depth realist synthesis [32] and a realist approach to thematic analysis [46]. This involved identification of how different strategies, mechanisms and contexts interact to produce particular outcomes resulting in the final programme theory. It included capturing data from qualitative discussions found in the included studies, describing how and why an intervention or parts of an intervention may or may not work and in what circumstances. Data on aspects of the study’s history and context, especially those highlighted as important by the study’s authors and any theories or mechanisms postulated (or assumed) by the study’s authors to explain the success or failure of the intervention, were also extracted. This information was tabulated in a Microsoft Excel spreadsheet and organised into CMOcs for each included study. From this, common overarching themes across the studies that contributed to the refined programme theory were identified. The articles were further re-read, and iteratively revised to capture additional themes or concepts that might contribute to the refined programme theory. Finally, an overall synthesis of these combinations of contexts, mechanisms and outcomes, independent of individual study details was conducted to generate the refined programme theory.

## Results

**Fig. 1:**
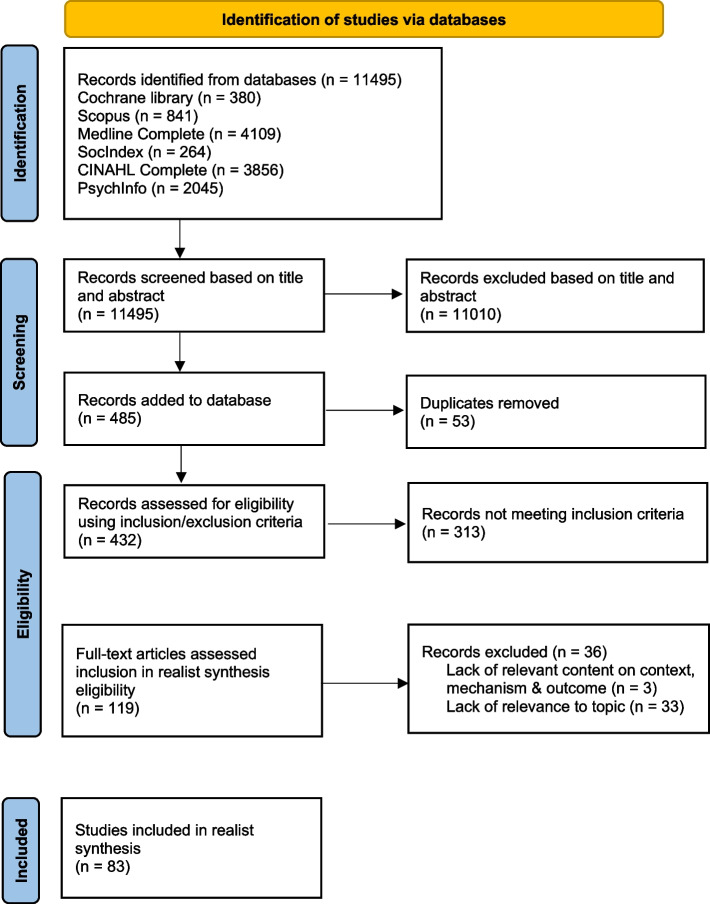
PRISMA flow diagram of study screening, selection, and inclusion

A total of 83 studies meeting the inclusion criteria and assessment for rigor and relevance were included. Study screening, eligibility, and selection processes are shown in Fig. 1.

### Initial programme theory

**Fig. 2:**
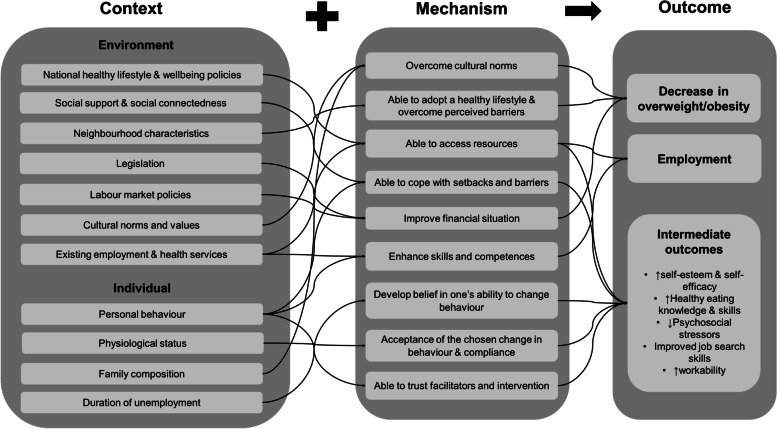
The initial program theory of how health-related interventions work to reduce obesity and unemployment, stated as context- mechanism-outcome configuration propositions and based on existing literature

Figure 2 illustrates the initial programme theory in terms of CMOc propositions based on brief initial review of the relevant literature, discussions and understanding drawn from professional experience. This process identified both individual and environmental factors to underlie the context of the interventions and how these interact with mechanisms to result in outcomes. This theory building was focused on key assumptions on how interventions designed to reduce overweight, obesity and unemployment work. Using our synthesis, we then set out to refine this initial program theory.

### Characteristics of included studies

Tables 1 and 2 present the summary and main findings of the studies included in this review. A total of 83 studies were included in this review and of these, 66.2% targeted overweight or obese participants and 33.7% unemployed individuals, jobseekers or trainees. 54.2% of included studies were randomised controlled trials (RCTs), 17 (20.5%) intervention studies, 19 (22.9%) quasi experimental studies, 1(1.2%) qualitative study and 1(1.2%) controlled study. The studies included were conducted in 24 countries with the majority (23.3%) in the USA, 14.0% in the United Kingdom, 12.8% in Australia and 49.9% in other countries including Germany, Finland, The Netherlands, Spain, Israel and Malaysia. Most studies (67.4%, *n* = 56) involved both male and female participants with age ranging from 18 to 64 years. Evaluation methods included both objective and subjective methods (45.8%), subjective methods only (44.6%) and objective methods only (8.4%). Reported outcomes included weight, BMI and other anthropometric measures [23, 53, 71, 73–75, 77, 79, 80, 82, 84, 85, 87, 89, 91–95, 98, 100, 101, 103–112, 114, 115, 117–120, 124], reemployment [22, 47, 52, 54, 57, 59, 61, 62, 65, 67–69], healthy eating knowledge and healthy eating behaviour [49, 56, 72, 74, 76, 78, 87, 98, 100, 110, 111, 113, 118–120, 124], self-efficacy and self-esteem [27, 48–51, 54, 56, 57, 59, 61, 66–70, 72, 75, 76, 78, 82, 86, 88, 90, 92, 96, 101, 102, 108, 112, 113, 118, 120, 121, 124], physical activity [20, 23, 74, 82–84, 89–91, 93, 96, 98, 104, 106, 107, 110, 111, 121, 124], job search and entrepreneurial skills [22, 55, 56] and wellbeing, mental and physical health [58, 59, 74, 83, 87, 97, 101, 121, 124].

**Table 1:** Summary of study characteristics and main findings of included studies targeted at participants who are unemployed

Author	Country	Target group	Sample size (n)	Intervention	Duration (Wks.)	Programme theory	Outcome
Brenninkmeijer et al.*,* 2011 [47]	The Netherlands	Low income/ Unemployed	118	Employment voucher/JOBS intervention	52	Not stated	26% participants reemployed
Britt et al.*,* 2016 [48]	Canada	Unemployed/ underemployed (< 20 hrs/week)	1434	Motivational interview & job search activities	Not stated	Transtheoretical model of change	63.0% participants reemployed
Chung et al.*,* 2019 [49]	Hong Kong	Construction trainees	36	Nutrition education	3	Transtheoretical model (TTM), stages of change	↑daily fruit consumption (P < 0.05) ↑daily vegetable consumption (*P* < 0.05), ↑healthy eating knowledge (P < 0.05). No change in healthy eating behaviour
Creed et al.*,* 2001 [50]	Australia	Unemployed	161	Occupational skills training	4-6	Deprivation Model/ Personal Agency theory	↑Improvement in job-search self-efficacy (P < 0.001) & self-esteem
Dambrun et al.*,* 2014 [27]	France	Unemployed	21	Positive psychology	2	Positive psychology	↓depression (*p* < 0.002) & anxiety (P = 0.05), ↑self-esteem (p < 0.05) No change in anxiety, self-efficacy, subjective fluctuating happiness
Eden et al.*,* 1993 [51]	Israel	Unemployed	66	Self-efficacy training/behavioural modelling	2.5	Motivation theory/Bandura’s theory of self-efficacy	62.5% participants remployed ↑ self-efficacy & job search activity.
Gabrys et al.*,* 2013 [20]	Germany	Unemployed	51	Physical activity counselling	12	5As approach (assess, advice, agree, assist, arrange)	↑ 9 minutes/day moderate-to- vigorous PA & 81 cpm total PA
González-Marín et al.*,* 2019 [52]	Spain	Unemployed	696	Job search and professional training	52	Not stated	47.3% of women & 40.7% of the men reemployed. No change in prevalence of poor perceived health. ↑improved mental health.
Harrell et al.*,* 1996 [53]	USA	Trainees	1504	Wellness and fitness programme	9	Not stated	↓ 5.6% in body fat
Hodzic et al.*,* 2015 [54]	Spain	Unemployed	73	Emotional competence training	0.4	Mayer and Salovey’s 4-branch model of emotional intelligence	21.2% participants reemployed ↑ perceived employability (P < 0.05) & entrepreneurial self-efficacy (*P* < 0.05). No changes in job search or entrepreneurial intention.
Hulshof et al.*,* 2020 [55]	The Netherlands	Unemployed	421	Job search training	6	Job-demand-resources theory, experiential learning theory	↑psychological capital & re-employment crafting. Positive effect on job search behaviour, goal setting & wellbeing. No effect on reemployment
Iseselo et al.*,* 2019 [56]	Tanzania	Unemployed	36	Health only/ entrepreneurship & health/ beekeeping & health or all three combined	65	Not stated	Participants acquired ability to establish sustainable business, increase in entrepreneurial skills, improved healthy lifestyle
Joseph et al.*,* 2001 [57]	USA	Unemployed	52	Self-generated imagery	2	Markus’s theory of possible selves	61.5% participants reemployed ↑self-esteem (P < 0.05) & perceived control (P < 0.05). ↓depression (P < 0.05),
Kreuzfeld et al.*,* 2013 [23]	Germany	Unemployed	119	Health competence & physical activity training	12	Not stated	↓percent body fat (*P* < 0.017). ↓depression (*P* < 0.028) No change in weight & BMI ↑physical activity
Limm et al.*,* 2015 [58]	Germany	Unemployed	287	Motivational interviewing	12	Not stated	Improved perceived mental and physical health scores. ↓anxiety score (−1.03, *P* = 0.012), No change in depression score
Malmberg-Heimonen et al.*,* 2005 [59]	Finland	Unemployed	672	Job-search training	12	Not stated	No change in re-employment ↓ depression in voluntary group. ↑Increased self-efficacy in voluntary group (*P* = 0.053)
Malmberg-Heimonen et al.*,* 2019 [60]	Finland	Unemployed	1015	Job search training	Not stated	Not stated	No change in reemployment
Noordzij et al.*,* 2013 [22]	The Netherlands	Unemployed	223	Learning-goal orientation training	2	Goal orientation theory/self-regulation	28% participants reemployed. ↑ job-search (P < 0.05) Positive effect on affected cognitive self-regulatory variables. No effect on self-efficacy
Proudfoot et al.*,* 1997 [61]	UK	Unemployed	209	Cognitive-behavioural training	7	Not stated	34% participants reemployed (*P* = 0.0006) ↑ GHQ score (P < 0.001), self-esteem (P = 0.01), job-seeking/self-efficacy (P = 0.001), motivation for work (P = 0.05), life satisfaction (*P* = 0.05) & attributional style (*P* = 0.001)
Reynolds et al.*,* 2010 [62]	Ireland	Unemployed	352	Job search & resilience training	0.2	Not stated	47.7% of participants reemployed(P < 0.001)
Robert et al.*,* 2019 [63]	France	Unemployed	704	Preventive medicine consultation	Not stated	Not stated	No change in reemployment status, no effect on social security or perceived health
Shirom et al.*,* 2008 [64]	Israel	Unemployed	442	Job-search & skill enhancement	1	Not stated	No effect on reemployment and self-efficacy
Stjernswärd et al.*,* 2013 [65]	Sweden	Unemployed	7	Rehabilitation & labour market training	10	Acceptance commitment therapy	↑ self-esteem & occupational aspiration. 6/7 participants reemployed or in training
van Ryn et al.*,* 1992 [66]	USA	Unemployed	308	Job-search skills or self-instructional material	2	Theory of planned behaviour; Theory of reasoned action	↑ job-search self-efficacy (P < 0.001)
Vastamäki et al.*,* 2009 [67]	Finland	Unemployed	74	Labour market activities, personal guidance & unemployment support	24	Sense of coherence theory	14.9% participants reemployed ↑ mean SOC (p < 0.01),
Vinokur et al.*,* 1995 [68]	USA	Unemployed	1801	Job search	1	Not stated	↑reemployment, self-esteem (p < 0.001), job-search self-efficacy (p < 0.001) & confidence in preparedness to handle setbacks (*p* < 0.001).
Vuori et al.*,* 1999 [69]	Finland	Unemployed	745	Labour market	1-24	Not stated	23.1% participants reemployed No change in psychological distress
Vuori et al.*,* 2005 [70]	Finland	Unemployed	1227	Job search	1	Social learning techniques	70.4% participants reemployed in a subsidized job, or in vocational training (p < 0.05). ↑self-esteem &↓depressive symptoms.

Abbreviations: *BMI* body mass index, *PA* physical activity, *SOC* sense of coherence, *TTM* Transtheoretical model

**Table 2:** Summary of study characteristics and main findings of included studies focused on individuals with overweight and obesity

Author	Country	Target group	Sample size (n)	Intervention	Duration (Wks.)	Programme theory	Main outcome
Ahern et al.*,* 2017 [71]	UK	BMI > 28 kg/m^2^	1267	Behavioural weight loss	12/52	Not stated	↓weight − 3.26 kg in brief intervention, −4.75 kg in the 12-week programme, −6.76 kg in the 52-week programme
Allicock et al.*,* 2010 [72]	USA	Overweight/obese, BMI ≥ 25 kg/m^2^	195	Motivational interviewing & nutrition education	24	Not stated	↑ of 1.7FV servings (P < 0.05)
Alves et al.*,* 2009 [73]	Brazil	Overweight/obese BMI ≥ 25 kg/m^2^	156	Aerobic exercise	24	Not stated	↑ weight − 1.69 kg, BMI, −0.63 kg/m^2^ (p < 0.001)
Aoun et al.*,* 2011 [74]	Australia	Overweight/obese BMI ≥ 27 kg/m^2^	40	Motivational interviewing	20	Not stated	↓ BMI Improvement in healthy dietary habits + Quality-of-life scores ↑ PA + 29 min/wk.
Ash et al.*,* 2006 [75]	Australia	Overweight/obese BMI ≥ 27 kg/m^2^	176	Cognitive behaviour therapy	8	Not stated	↓weight − 2.8 kg (P < 0.05). No change in body fat percent; No change in physical activity. ↑ self-efficacy scores (*P* = 0.02)
Azar et al.*,* 2018 [76]	Iran	Obese BMI ≥ 30 kg/m^2^	30	Group schema therapy	8	Not stated	↓concern about weight, diet (p < 0.001) and negative physical self-concept (*p* < 0.001).
Beatty et al.*,* 2020 [77]	USA	Overweight/obese BMI 25-40 kg/m^2^	72	Self-monitoring device	8	Social cognitive theory	↓weight 0.8 kg (*P* = 0.003)
Beintner et al.*,* 2019 [78]	Germany	Overweight/obese BMI > 25 kg/m^2^	323	Health promotion	12	Not stated	No change in weight. ↑1.15 portions in FV consumption (P < 0.001). ↑ self-esteem (*P* < 0.001) & life satisfaction (p < 0.001)
Benyamini et al.*,* 2013 [79]	Israel	Overweight/obese BMI > 27 kg/m^2^	632	Structured intentions and action planning	10	Not stated	↓ BMI −1.10(IIC), 1.11(BIC)
Berg et al.*,* 2008 [80]	Germany	Obese BMI 30-40 kg/m^2^	517	Lifestyle modification	52	Not stated	↓in weight − 6.4 kg (P < 0.001), BMI −2.2 kg/m^2^ in BMI (*P* < 0.001) & WC −7.2 cm (P < 0.001)
Berli et al.*,* 2016 [81]	Switzerland	Overweight/obese BMI ≥ 25 kg/m^2^	121	Physical activity	2	Action control	No change in PA
Bouhaidar et al.*,* 2013 [82]	USA	Overweight/obese BMI 25–40 kg/m^2^	26	SMS behaviour modification	12	Health Promotion model	↓weight (*P* = 0.047) No change in eating behaviours (*P* = .06); exercise and nutrition self-efficacy (P = .06); ↑PA total MET-minutes/wk.;
Breslin et al.*,* 2019 [83]	Ireland	Overweight/obese BMI > 25 kg/m^2^	49	Physical activity	6	Not stated	↓ weight (−3.74 kg, P < 0.001), anxiety score (−4.56, P < 0.001), social dysfunction score (− 3.64, P < 0.001), GHQ depression score (− 2.96) ↑ PA pedometer scores (+ 31,335.11, P < 0.001)
Brumby et al.*,* 2013 [84]	Australia	Overweight/ obese BMI ≥ 25 kg/m^2^	68	Physical activity	24	Not stated	↓ -2.64 kg (p < 0.001), WC − 2.01 (p = 0.02) & BMI −0.97 kg/m^2^ (P < 0.001). No change in waist-to-hip ratio, body fat percentage and DASS total score ↑ PA 94.4%
Collins et al.*,* 2012 [85]	Australia	Overweight/obese BMI 25-40 kg/m^2^	309	Behaviour change	12	Social cognitive theory	↓ weight in enhanced (− 2.98) & basic (− 2.14 kg) intervention. ↓BMI in enhanced (−0.98 kg/m^2^) and basic (− 0.72 kg/m2) intervention & ↓WC. No change in PA & quality of life. ↓energy intake (*p* = 0.03)
Chung et al.*,* 2014 [86]	Hong Kong	Overweight/obese BMI ≥ 25 kg/m^2^	60	Nutrition education & electronic dietary recording system	12	Not stated	↑ dietary recommendation knowledge in the EG (*p* = 0.009) and FD groups (*p* = 0.046), eating attitudes scores FD group (*p* = 0.017). No change HPALwork, sport or leisure indices
Cleo et al.*,* 2019 [87]	Australia	Overweight/obese (BMI) ≥ 25 kg/m^2^	75	Habit-based lifestyle	12	Not stated	↓ -2.4 kg in TTT group, − 1.7 kg DSD group. ↓ BMI − 0.81 kg/m^2^ TTT group, − 0.6 kg/m^2^ DSD group, WC − 3.1 cm TTT group, − 2.0 cm DSD group. + healthy behaviour, depression and anxiety and in habits and depression
Dallow et al.*,* 2003 [88]	USA	Obese BMI > 30 kg/m^2^	44	Physical activity	24	Transtheoretical model, self-efficacy theory	↑ self-efficacy (*P* = 0.016) ↑ energy expenditure
Dean et al.*,* 2018 [89]	USA	Overweight/obese BMI > 25 kg/m^2^	34	Physical activity	10	Self-determination theory, social ecological framework of health behaviour, social cognitive theory	↑ PA (P < 0.05). ↓weight (P < 0.05), body fat percentage − 1.2%, *P* < 0.05) No change in BMI, WC
del Rey-Moya et al.*,* 2012 [90]	Spain	Obese BMI > 30 kg/m^2^	130	Physical activity	7	Not stated	No change in weight, BMI, WC ↑number of hours spent walking (*P* = 0.007) & PA hours (P = 0.009)
Dombrowski et al.*,* 2012 [91]	UK	Obese BMI > 35 kg/m^2^	74	Dietary and physical activity behaviour change	5	Self-regulation theory, social cognitive theory, social comparison theory, relapse prevention model	↓ weight − 0.86 kg (*P* = 0.0001) ↑ of 1.6 PA sessions (*P* = 0.002) No change in diet.
Folta et al.*,* 2009 [92]	USA	Overweight/obese BMI > 24 kg/m^2^	96	Physical activity	12	Social cognitive theory	↓ weight (−2.1 kg), WC (− 2.3in), BMI (−0.8 kg/m^2^) energy intake (−390 kcal/d). ↑ PA (+ 1637 steps/day), dietary & PA self-efficacy scores
Garcia et al.*,* 2019 [93]	USA	Overweight/obese BMI 25-50 kg/m^2^	50	Diet and physical activity	12 /24	Social cognitive theory, problem solving theory	↓weight (−6.3 kg), body fat percent (−1.6%), WC (−4.7 cm). ↑PA 183 minutes/week. ↓ mean caloric dietary intake (−51.3%)
Godino et al.*,* 2019 [94]	USA	Obese/overweight BMI 27-39.9 kg/m^2^	298	Personalised text message & health-coaching	52	Not stated	↓weight − 1.68 (−3.08 to −0.27) in ConTxt only, & − 3.63 (−5.05 to −2.81) in ConTxt plus health-coaching calls.
Gram et al.*,* 2014 [95]	Denmark	Overweight/obese BMI 25-30 kg/m^2^	6	Physical activity	12	Theory of planned behaviour	↓weight (−3.8 kg) for moderate exercise group (−2.2 kg) for high exercise. ↓BMI in moderate and high exercise groups
Grey et al.*,* 2019 [96]	UK	Overweight/obese, BMI 25-40 kg/m2	59	Physical activity	12	Evolutionary mismatch	↑PA (p < 0.05) ↓ energy intake (− 431 kcal/day, p < 0.01)
Groh et al.*,* 2015 [97]	USA	Overweight/obese, BMI ≥ 30/WC >35in	55	Nutrition education & physical activity	24	Not stated	↑Mental component summary score (p < 0.001).
Hardcastle et al.*,* 2008 [98]	UK	Overweight/obese, BMI ≥28 kg/m2	218	Nutrition and physical activity education	24	Not stated	↑ walking (114 min/week, p = 0.01), combined PA (p = 0.05) ↓BMI (p = 0.01) ↑FV intake, ↓fat intake
Hardcastle et al.*,* 2013 [99]	UK	Overweight/obese, BMI ≥ 28	334	Motivational interviewing	24	Self-determination theory	↑walking at 6 months (*p* = 0.006) & 18 months (*p* = 0.032) No change in dietary fat intake No change in BMI
Hutchesson et al.*,* 2014 [100]	Australia	Overweight/obese BMI 25-40 kg/m^2^	268	Behaviour change	12	Social cognitive theory	↓weight − 2.3 kg (basic), −3.1 kg (enhanced), P < 0.001) ↑ percentage of energy contribution from fruits and reduced energy-dense, nutrient-poor foods (P < 0.001)
Jane et al.*,* 2017 [101]	Australia	Overweight/obese BMI 25-40 kg/m^2^	67	Nutrition education & physical activity	24	Not stated	↓weight (p = 0.016), WC (P = 0.01) + psychological health (*p* = 0.022) No change in energy intake & PA
Kegler et al.*,* 2016 [102]	USA	Overweight/obese	349	Improvement of home environment	16	Social-cognitive theory	↓ energy intake (− 274 kcal) No change in PA
Keller et al.*,* 2001 [103]	USA	Overweight/obese, BMI > 25 kg/m^2^	36	Physical activity	24	Not stated	↓ weight (−1.36 kg) & BMI(−1 kg/m^2^) in low frequency group ↑ weight(+ 1.36 kg) in high frequency group
Kleist et al.*,* 2017 [104]	Germany	Overweight/obese, BMI 27-35 kg/m^2^	82	Energy restricted diet &physical activity	12	Not stated	↓weight (−8.8 kg), total fat mass (−6.4), BMI (−2.8 kg/m^2^) ↑PA (4.6MET-h/24 h) in DI + walking group. ↓weight (−7.0 kg), BMI (− 2.3 kg/m^2^), fat mass (−4.8 kg) ↑PA (0.5 MET-h/24 h) in diet only group.
Kraushaar et al.*,* 2014 [105]	Germany	Overweight/obese BMI > 25 kg/m^2^	82	Physical activity & behaviour change	24	Adoption of cognitive feedback control	+ VO2 peak of 3.7 ml/kg/min ↓BMI (−1.6 kg/m^2^), weight (−4.8 kg) and fat mass (−3.6 kg)
Lee et al.*,* 2011 [106]	South Korea	Obese/overweight BMI ≥ 25 kg/m^2^	49	Physical activity, behaviour change & nutrition education	12	Self-management	↓BMI (−1.05, p < 0.001) in self-management group, (− 1.22, *p* < 0.001) in structured exercise group. ↑Total exercise time by > 20 min in each exercise session (*p* = 0.005) self-management group (p < 0.001) structured exercise group.
Lutes et al.*,* 2010 [107]	USA	Overweight/obese BMI 31.4 kg/m^2^	25	Behaviour change	12	Small changes approach/Problem solving Therapy (PST)	↓weight (−3.2 kg, p < 0.001), BMI (− 1.2 kg/m^2^, p < 0.001) ↑ daily step count (*p* = 0.08) No change in caloric intake
Marquez et al.*,* 2013 [108]	USA	Overweight/obese BMI 27-50 kg/m^2^	27	Behaviour change	12	Not stated	↓ weight in both groups (ILG: −4.7 kg & PLG: − 4.3 kg) ↑weight loss self-efficacy (p < 0.01), exercise self-efficacy (p = 0.02), family social support for exercise habits (p = 0.01) No changes in PA (*p* = 0.59)
Mayer et al.*,* 2019 [109]	USA	Overweight/obese BMI ≥25 kg/m^2^	402	Behaviour change	24	Not stated	↓percentage weight (− 1.4%, *p* = 0.008) ↑ BMI (+ 0.007 kg/m^2^) No changes in FV intake
McRobbie et al.*,* 2019 [110]	UK	Overweight/obese BMI ≥ 28 kg/m^2^	295	Dietary, physical activity & behaviour change	8	Not stated	↓weight (−4.2 kg) in WAP arm than in PNI arm (−2.3 kg) ↑knowledge of caloric content of food. ↑PA (359 in WAP vs. 215 in MET-minutes/week, in PNI, p = 0·18).
Mohamed et al.*,* 2018 [111]	Malaysia	Overweight/obese BMI > 23 kg/m^2^	61	Dietary, physical activity & behaviour change	12	Not stated	↑ vegetable intake (+ 1.0 serving size). ↓total calorie intake 9-375 kcal/day). ↑PA (+2366MET-minutes/week) ↓weight (−2.5 kg), BMI (−1.2 kg/m^2^) body fat percentage (− 1.6%).
Mohd et al.*,* 2017 [112]	Malaysia	Overweight/obese 25.0 - 39.9 kg/m^2^	209	Dietary, physical activity & behaviour change	52	Not stated	↓weight (−1.13 kg, p < 0.05) No change in BMI.
Mummah et al.*,* 2017 [113]	USA	Overweight/obese BMI 28-40 kg/m^2^	135	Mobile app behaviour change	12	Social Cognitive Theory	↑ + 2 servings vegetables (p = 0.04)
Park et al.*,* 2009 [114]	South Korea	Overweight/Obese BMI > 23 kg/m^2^	49	Nutrition education	8	Not stated	↓weight (−1.6 kg; p < 0.05), WC (−2.8 cm; *p* < 0.05).
Silina et al.*,* 2017 [115]	Latvia	Overweight/obese BMI > 25 kg/m^2^	123	Dietary & behaviour change	52	Planned behavioural theory and social cognitive theory	↓weight (−2.4 kg), BMI (−0.81 kg/m^2^), WC (−5.0 cm)
Sniehotta et al.*,* 2019 [116]	UK	Overweight/obese BMI ≥30 kg/m^2^	264	Behaviour change	52	Self-regulation theory	No change in weight (−0.07 kg, *p* = 0.9)
Solbrig et al.*,* 2018 [117]	UK	Overweight/obese BMI ≥25 kg/m^2^	114	Functional Imagery Training or Motivational Interviewing	24	Elaborated Intrusion theory; Motivational Interviewing	↓weight (−4.11 kg, p < 0.001), WC (−7.02 cm, p < 0.001)
Tapsell et al.*,* 2014 [118]	Australia	Overweight/obese BMI 25-35 kg/m^2^	113	Dietary	52	Not stated	↓weight (−6.5 kg) and energy intake (−2000kj/day, p < 0.001).
Tapsell et al.*,* 2016 [119]	Australia	Overweight/obese BMI 25-40 kg/m^2^	21	Diet & physical activity behaviour change	12	Acceptance commitment theory	↓weight (−3.98 kg, p = 0.002), BMI (−1.24 kg/m^2^, p = 0.002), body fat percent (− 3.25%, *p* = 0.034), WC (5.14 cm, p = 0.001) ↓energy from dietary fat (− 4.5%, *p* = 0.004). No change in quality of life & PA
Uemura et al.*,* 2019 [120]	Japan	Overweight/obese BMI ≥ 25 kg/m^2^	44	Nutrition education	8	Not stated	↓weight (−1.69 kg, p < 0.001), BMI (−0.71 kg/m^2^, p < 0.001), WC (− 1.91 cm, p < 0.001) ↑dietary fibre intake (p < 0.001) ↓CES-D score.
Watkins et al.*,* 2014 [121]	USA	Overweight/obese Average BMI 34.4	38	Physical activity & behaviour change	12	Not stated	No change in weight, BMI or body fat percentage. ↑PA score (p < 0.001) + depression scores (p < 0.02)
Whitelock et al.*,* 2019 [122]	UK	Overweight/obese BMI ≥25.0 kg/m^2^	107	Dietary education	8	Not stated	No change in weight, energy intake and self-efficacy
Whitham et al.*,* 2014 [123]	UK	Overweight/obese BMI 27-35 kg/m^2^	85	Dietary intervention/education	12	Not stated	No change in weight
Wyke et al., 2019 [124]	England, The Netherlands, Norway & Portugal	Overweight/obese BMI ≥27 kg/m^2^	1113	Physical activity, diet &behaviour change	12	Self-determination theory	↓ weight (−2.6 kg, *p* < 0.0001), BMI (−0.8 kg/m2, p < 0.0001), WC (−3.3 cm, p < 0.0001). ↑PA (mean step count of + 678 steps/day, p < 0.001) + in wellbeing, self-esteem & dietary intake No change in quality of life
Young et al.*,* 2015 [125]	Australia	Overweight/obese BMI 25-40 kg/m^2^	92	Physical activity & nutrition education	52	Social cognitive theory	No change in PA & discretionary food cognitions.

Abbreviations: *BIC* behavioural intentions condition, *BMI* body mass index, *CES-D* Centre for Epidemiologic Studies-Depression Scale, *DASS* Depression and Anxiety Stress Scale, *ED* electronic diary, *FD* food diary, *FV* fruit and vegetables, *GHQ* General Health Questionnaire, *HPAL* Habitual Physical Activity Level, *IIC* implementation intentions condition, *ILG* individual Lifestyle Group, *MET* Metabolic Equivalent of Task, *PA* physical activity, PLG; *PNI* practice nurse intervention, *WAP* Weight Action Programme, *WC* waist circumference

### Common approaches used in interventions designed to reduce overweight, obesity and unemployment in adults

Intervention strategies that were commonly used by studies to address obesity and unemployment were identified and categorised as follows: (i) building knowledge and skills to enable behaviour change [20, 22, 23, 49, 53, 55, 56, 60, 63, 64, 68, 69, 71, 72, 74, 75, 77–81, 83–87, 89, 92, 93, 96, 98, 99, 101–116, 118–120, 122, 124, 125], (ii) increasing motivation [48, 58, 67, 72, 74, 88, 89, 99, 113, 117, 119, 124] (iii) cognitive behaviour therapy/positive psychology [27, 61, 65, 75, 76], (iv) improving self-efficacy, confidence and self-esteem [47, 50, 51, 59, 62, 66, 67, 75, 79, 85, 88, 89] (v) building resilience and emotional competency [51, 54, 57, 59, 62, 66–68, 121], hands-on practice of behaviour [20, 52, 53, 68, 71, 77–81, 83–85, 87, 90–92, 95, 100, 101, 103, 105, 106, 108, 110–113, 116, 119, 121, 125] and (vii) building knowledge and skills on goal-setting, identifying barriers to achieving goals, and self-monitoring [74, 77–79, 82, 91, 93, 94, 96, 100, 102, 107, 108, 113, 118, 125]. The majority of studies used more than one strategy in the delivery of interventions.

### Factors underlying the success or failure of interventions

Factors that contributed to the success of interventions included: longer length of intervention [71], more contact time with participants [65, 110, 114, 119], culturally or gender tailored intervention [52, 72, 75, 83, 93, 94, 99, 102, 107–109, 113, 114, 119, 124], regular monitoring and support [20, 51, 54, 55, 62, 75, 88, 89, 93, 97, 103, 104, 106], positive attitude of coaches [74], simplicity of tasks/messages [66, 82, 84, 85, 94, 108, 115, 119, 120], high satisfaction and acceptance of intervention [22, 58, 68, 106, 117, 121], variation in activities [56, 88], interactive and engaging activities [58, 86, 89, 94, 96, 101, 113], small changes approach [96, 107] and high compliance [95, 104, 105, 113, 115]. Factors that reduced the effectiveness of interventions included poor adherence/low compliance [90, 99, 122], lack of specificity and clarity in intervention goals [96, 124], low participation rate [64, 98, 125], short duration of intervention [71, 100], minimal contact, lack of structure and follow-up [56, 63, 97, 116] and intervention not tailored to the individual [64, 81]. Participant characteristics that influenced the success or failure of the interventions included age [49, 58, 63, 68, 78, 89, 99, 124], gender [58, 63, 64, 68], length of unemployment [58], income level, educational level, baseline BMI, self-efficacy and self-esteem [50, 51, 78, 79, 96, 124], motivation [95] and availability of social support [52].

### Refined Programme theory

A total of 8 CMOCs were generated building up on the initial programme theory. These are as follows (the letter, C-context, M-mechanism and O-outcomes). The CMOcs provide a higher level of abstraction that sets out the underpinning logic behind the family of interventions strategies identified to address unemployment and obesity.

1. **CMO1:** When participants with limited knowledge about healthy eating (**C**) are provided with the requisite knowledge and skills, and able to apply these new knowledge and skills (**M**), their healthy eating behaviour is improved (**O**).
2. **CMO2:** When participants with low educational status (**C**) are provided with an intervention delivered in their native language, there is higher acceptance, and they are able to utilise the new skills to successfully execute new behaviour (**M**) and will improve healthy eating behaviour (**O**).
3. **CMO3:** When participants are provided with healthy eating and physical activities tailored to their needs (**C**), they are able to incorporate skills and strategies into daily routine, successfully execute new skills (**M**) and reduce their weight and BMI (**O**).
4. **CMO4:** When participants with low income (**C**) are provided with financial incentives and resources, they are able to purchase healthier food options (**M**) and will improve their healthy eating behaviour (**O**).
5. **CMO5:** When participants receive healthy eating and physical activity interventions in group settings (**C**), they are able to obtain social support from peers (**M**) and will increase their physical activity levels and improve healthy eating behaviours (**O**).
6. **CMO6:** When participants with limited knowledge and job search skills (**C**) are provided with job search skills training, they are able to apply these skills in their job search (**M**) and will obtain employment (**O**).
7. **CMO7:** When labour market conditions are favourable (**C**) and participants are provided with job search and entrepreneurial skills training, participants are able to develop and apply their new employability skills (**M**) and will obtain employment (**O**).
8. **CMO8:** When participants with low motivation and self-esteem (**C**) are offered self-led interventions, they will be able to develop self-regulatory skills, maintain perceptions of control over situation (**M**) and improve their self-efficacy and self-esteem (**O**).

## Discussion

To our knowledge, this review represents the first use of realist synthesis to understand the determinants of the effectiveness of complex health-related interventions to reduce overweight, obesity and unemployment. Building on our initial programme theory and exploring the interactions between the contexts of the interventions, mechanisms, intervention strategies and outcomes, a number of key insights were obtained. The most common intervention strategy used by the majority of studies was knowledge and skills building through provision of workshops, lectures, information leaflets or skills training. This approach was often based on assumptions that participants lacked the requisite knowledge or skills to be able to implement healthy eating behaviour or obtain jobs. While this strategy resulted in mixed successes, more positive outcomes were observed when participants had low educational status, lower income, or when the intervention implemented tailored and culturally appropriate activities (CMO1, CMO2, CMO6). This approach enabled the acquisition of skills relevant to participants’ needs thereby facilitating the incorporation of these new skills into daily routine and increased the ability to successfully execute and maintain the new behaviour.

Evidence from research show that there is no universal model of an intervention that results in positive outcomes for all participants [126]. For example, individuals who are unemployed may have varied level of skills and overweight or obese may have different underlying determinants, therefore interventions need to be tailored to individual needs [55, 119, 126, 127]. Our synthesis indicated that age, gender, baseline educational level, BMI, self-efficacy, self-esteem and motivation impacted the success or failure of the intervention [49, 67, 71, 85, 102, 112]. Tailored activities led to higher acceptance, compliance, participation rate and satisfaction [22, 95, 104, 106]. Additionally, resources are wasted and opportunities to provide genuine help are lost if an intervention is not appropriate to the needs of an individual or the targeted group [127].

However, there is limited evidence about the cost-effectiveness of tailored interventions compared to generalised interventions. In addition, there is insufficient evidence on the most effective approaches to tailoring, including how determinants should be identified, how decisions should be made on which determinants are most important to address, and how interventions should be selected to account for the important determinants. This highlights a need for programmes co-produced with participants using participatory approaches to prioritise the needs of the target group thereby making them more meaningful and engaging.

Another key context that impacted the effectiveness of interventions was delivery of activities in group-based or individualised or self-led contexts (CMO5). Group programmes offer a more cost-effective option to individual programmes [101] and can serve as an important source of vicarious learning and social support [89]. The effectiveness may however be dependent on the demography of participants (age group, gender, culture) or sensitivity of intervention elements. In a previous study involving African American men, participants enjoyed the camaraderie and support they received from their small group and benefitted from seeing that others were struggling with and overcoming similar barriers to physical activity they faced [89]. The men in this study reported that they learned from and supported one another with strategies to overcome barriers to physical activity. On the contrary, anxiety and discomfort in group settings as well as reticence to engage in activities appeared to be a frequent issue for group-based interventions [65] and group dynamics could significantly influence uptake of activities [91]. It is therefore critical that programmes consider what works for the target population.

Other factors that accounted for success of interventions implemented to reduce weight and unemployment included, multicomponent programme activities, favourable labour market conditions (CMO7), demographic characteristics of target population and provision of financial incentives or other resources that enabled hands-on practice of behaviour (CMO4). Evidence from the literature show that interventions which had varied, diverse and engaging activities had a higher uptake and compliance leading to positive outcomes [101, 126]. For example, it is essential to combine measures for changes in nutrition, physical activity, and behaviour in interventions seeking to reduce overweight and obesity [128]. Furthermore, programmes that focus on a healthy lifestyle by concurrently offering dietary advice with behavioural strategies such as increasing physical activity are more effective than programs that focus on dietary restriction alone [83, 129], suggesting a holistic lifestyle approach is warranted. Similarly, being unemployed denies people from the manifest (income) and latent (e.g., time structure, status, and identity) benefits of having a job, therefore, to optimise the effectiveness of interventions supporting the unemployed, a combination of job search skills training, enhancing coping skills and motivational approaches are required [54, 55]. Successful re-employment has been shown to depend on favourable conditions in the labour market, demographic characteristics (e.g., age, gender, educational attainment), and occupational characteristics (e.g., an academic degree). Young age and high level of education are positively related to reemployment [64]; therefore, programmes need to take these contexts into account during intervention design and implementation. Finally, a key finding from this review relates to the similarities in targeting common underlying factors such as low self-efficacy and self-esteem, low socioeconomic status, low skills and psychosocial stressors for both employment and heathy weight interventions. Implementing interventions that addressed these common underlying factors as well as psychological mechanisms assumed to regulate weight and unemployment, resulted in positive weight and employment outcomes. While addressing these underlying factors may contribute to improving employability and maintaining a healthy weight, further research is warranted to elucidate the extent to which these factors are moderated by the different interventions. Furthermore, it is important to highlight that unemployment and obesity are very complex conditions, with equally complex interacting mechanisms and contexts, therefore the CMOCs identified also indicate a degree of interconnectedness and the likely potential of interactions in other to achieve successful and effective interventions.

### Strengths and limitations

Our use of the realist approach of configuring contexts and mechanisms together is a key strength and adds explanatory power to help us understand how these elements interact to produce outcomes of interest in health-related interventions to reduce obesity and unemployment. Importantly, obesity and unemployment are very complex issues, and the use of realist review methodology enabled us to identify the complexity within the interventions as well as the multiple interactions between the numerous components of the implemented programmes.

The strength of the findings in this synthesis are also dependent on the comprehensiveness of the information provided on intervention contexts, mechanisms and outcomes. The majority of studies on health-rated interventions and therefore included in this synthesis were RCTs, which present a major limitation for this review. Characteristic of RCTs, there is attribution of success of interventions to randomisation and the actual programme without elucidation of why intervention was successful or the mechanisms underlying participants’ response to an intervention. There was also a lack of subgroup analyses in the majority of the studies, thus outcomes which may in fact be explained by differences among individuals were attributed to the intervention and this limited the identification of who the interventions worked for. Finally, the CMOcs identified in this review not exhaustive but rather an insight into what may be contributing to positive or negative outcomes and how certain determinants can be incorporated to achieve the desired outcomes therefore further exploration of the possible causal pathways are warranted.

## Conclusions

This review was able to identify contextual mechanisms that determined observed outcomes and how those involved in health-related interventions to reduce obesity and unemployment tended to respond to the intervention. It also uncovered a number of overlooked perspectives which should be included in future research. Multicomponent interventions combining different strategies, tailored to participants, using a mix of knowledge and skill building, motivational approaches and hands-on practice resulted in positive outcomes. Participant characteristics that influenced the outcomes included age, gender, educational status, income level and these should be considered when tailoring interventions. Taken together, this review contributes to an emerging field in systematic review, in which qualitative approaches compliment and extend the findings of quantitative reviews and highlights a co-produced rather than prescriptive approach to the design and implementation of health-related interventions to reduce overweight, obesity and unemployment.

## Supplementary Information

**Additional file 1.** Supplementary data.

## Data Availability

Not applicable – Realist systematic review of published studies.

## Abbreviations

CMO: Context-Mechanism-Outcome
RCT: randomised controlled trial
