# Supplementary material for: Systematic realist synthesis of health-related and lifestyle interventions designed to decrease overweight, obesity and unemployment in adults
Source: BMC Public Health. 2022 Nov 17;22:2100. doi: 10.1186/s12889-022-14518-6 (PMC9668709; doi:10.1186/s12889-022-14518-6)
Supplement: Supplementary file 1 — Additional file 1. Supplementary data. [file 12889_2022_14518_MOESM1_ESM.docx]

**SUPPLEMENTARY DATA**

**Supplementary Table S1**: RAMESES checklist of items to be included when reporting a realist synthesis

| **Checklist item** | | | **Location where item is reported Page** |
| --- | --- | --- | --- |
|  | TITLE |  |  |
| 1 |  | In the title, identify the document as a realist synthesis or review | 1 |
| ABSTRACT | | |  |
| 2 |  | While acknowledging publication requirements and house style, abstracts should ideally contain brief details of: the study's background, review question or objectives; search strategy; methods of selection, appraisal, analysis and synthesis of sources; main results; and implications for practice. | 2 |
| INTRODUCTION | | |  |
| 3 | Rationale for review | Explain why the review is needed and what it is likely to contribute to existing understanding of the topic area. | 5-6 |
| 4 | Objectives and focus of review | State the objective(s) of the review and/or the review question(s). Define and provide a rationale for the focus of the review. | 6 |
| METHODS | | |  |
| 5 | Changes in the review process | Any changes made to the review process that was initially planned should be briefly described and justified. | N/A |
| 6 | Rationale for using realist synthesis | Explain why realist synthesis was considered the most appropriate method to use. | 7 |
| 7 | Scoping the literature | Describe and justify the initial process of exploratory scoping of the literature. | 8 |
| 8 | Searching processes | While considering specific requirements of the journal or other publication outlet, state and provide a rationale for how the iterative searching was done. Provide details on all the sources accessed for information in the review. Where searching in electronic databases has taken place, the details should include, for example, name of database, search terms, dates of coverage and date last searched. If individuals familiar with the relevant literature and/or topic area were contacted, indicate how they were identified and selected. | 8-10 |
| 9 | Selection and appraisal of documents | Explain how judgements were made about including and excluding data from documents and justify these. | 8-10 |
| 10 | Data extraction | Describe and explain which data or information were extracted from the included documents and justify this selection. | 10 |
| 11 | Analysis and synthesis processes | Describe the analysis and synthesis processes in detail. This section should include information on the constructs analysed and describe the analytic process. | 11- |
| RESULTS | | |  |
| 12 | Document flow diagram | Provide details on the number of documents assessed for eligibility and included in the review with reasons for exclusion at each stage as well as an indication of their source of origin (for example, from searching databases, reference lists and so on). You may consider using the example templates (which are likely to need modification to suit the data) that are provided. | 11, Figure 1 |
| 13 | Document characteristics | Provide information on the characteristics of the documents included in the review. | 12, Tables 1-2 |
| 14 | Main findings | Present the key findings with a specific focus on theory building and testing. | 13-15 |
| DISCUSSION | | |  |
| 15 | Summary of findings | Summarize the main findings, taking into account the review's objective(s), research question(s), focus and intended audience(s). | 15-18 |
| 16 | Strengths, limitations and future research directions | Discuss both the strengths of the review and its limitations. These should include (but need not be restricted to) (a) consideration of all the steps in the review process and (b) comment on the overall strength of evidence supporting the explanatory insights which emerged. The limitations identified may point to areas where further work is needed. | 18-19 |
| 17 | Comparison with existing literature | Where applicable, compare and contrast the review's findings with the existing literature (for example, other reviews) on the same topic. | 18-19 |
| 18 | Conclusion and recommendations | List the main implications of the findings and place these in the context of other relevant literature. If appropriate, offer recommendations for policy and practice. | 19 |
| 19 | Funding | Provide details of funding source (if any) for the review, the role played by the funder (if any) and any conflicts of interests of the reviewers. | 20 |

**Supplementary table S2:** PRISMA Checklist

| **Section and Topic** | **Item #** | **Checklist item** | **Location where item is reported** |
| --- | --- | --- | --- |
| **TITLE** | | |  |
| Title | 1 | Identify the report as a systematic review. | 1 |
| **ABSTRACT** | | |  |
| Abstract | 2 | See the PRISMA 2020 for Abstracts checklist. | 2 |
| **INTRODUCTION** | | |  |
| Rationale | 3 | Describe the rationale for the review in the context of existing knowledge. | 4-6 |
| Objectives | 4 | Provide an explicit statement of the objective(s) or question(s) the review addresses. | 6 |
| **METHODS** | | |  |
| Eligibility criteria | 5 | Specify the inclusion and exclusion criteria for the review and how studies were grouped for the syntheses. | 9-10 |
| Information sources | 6 | Specify all databases, registers, websites, organisations, reference lists and other sources searched or consulted to identify studies. Specify the date when each source was last searched or consulted. | 8 |
| Search strategy | 7 | Present the full search strategies for all databases, registers and websites, including any filters and limits used. | Supplementary table S3 |
| Selection process | 8 | Specify the methods used to decide whether a study met the inclusion criteria of the review, including how many reviewers screened each record and each report retrieved, whether they worked independently, and if applicable, details of automation tools used in the process. | 8, Figure 1 |
| Data collection process | 9 | Specify the methods used to collect data from reports, including how many reviewers collected data from each report, whether they worked independently, any processes for obtaining or confirming data from study investigators, and if applicable, details of automation tools used in the process. | 8-10 |
| Data items | 10a | List and define all outcomes for which data were sought. Specify whether all results that were compatible with each outcome domain in each study were sought (e.g. for all measures, time points, analyses), and if not, the methods used to decide which results to collect. | 10, Tables 1-2 |
|  | 10b | List and define all other variables for which data were sought (e.g. participant and intervention characteristics, funding sources). Describe any assumptions made about any missing or unclear information. | 10 |
| Study risk of bias assessment | 11 | Specify the methods used to assess risk of bias in the included studies, including details of the tool(s) used, how many reviewers assessed each study and whether they worked independently, and if applicable, details of automation tools used in the process. | 10 |
| Effect measures | 12 | Specify for each outcome the effect measure(s) (e.g. risk ratio, mean difference) used in the synthesis or presentation of results. | Not applicable |
| Synthesis methods | 13a | Describe the processes used to decide which studies were eligible for each synthesis (e.g. tabulating the study intervention characteristics and comparing against the planned groups for each synthesis (item #5)). | Not applicable |
|  | 13b | Describe any methods required to prepare the data for presentation or synthesis, such as handling of missing summary statistics, or data conversions. | 11-12 |
|  | 13c | Describe any methods used to tabulate or visually display results of individual studies and syntheses. | Not applicable |
|  | 13d | Describe any methods used to synthesize results and provide a rationale for the choice(s). If meta-analysis was performed, describe the model(s), method(s) to identify the presence and extent of statistical heterogeneity, and software package(s) used. | 11 |
|  | 13e | Describe any methods used to explore possible causes of heterogeneity among study results (e.g. subgroup analysis, meta-regression). | Not applicable |
|  | 13f | Describe any sensitivity analyses conducted to assess robustness of the synthesized results. | Not applicable |
| Reporting bias assessment | 14 | Describe any methods used to assess risk of bias due to missing results in a synthesis (arising from reporting biases). | Not applicable |
| Certainty assessment | 15 | Describe any methods used to assess certainty (or confidence) in the body of evidence for an outcome. | Not applicable |
| **RESULTS** | | |  |
| Study selection | 16a | Describe the results of the search and selection process, from the number of records identified in the search to the number of studies included in the review, ideally using a flow diagram. | Figure 1 |
|  | 16b | Cite studies that might appear to meet the inclusion criteria, but which were excluded, and explain why they were excluded. | Not applicable |
| Study characteristics | 17 | Cite each included study and present its characteristics. | Tables 1-2 |
| Risk of bias in studies | 18 | Present assessments of risk of bias for each included study. | Not applicable |
| Results of individual studies | 19 | For all outcomes, present, for each study: (a) summary statistics for each group (where appropriate) and (b) an effect estimate and its precision (e.g. confidence/credible interval), ideally using structured tables or plots. | Tables 1-2 |
| Results of syntheses | 20a | For each synthesis, briefly summarise the characteristics and risk of bias among contributing studies. | Not applicable |
|  | 20b | Present results of all statistical syntheses conducted. If meta-analysis was done, present for each the summary estimate and its precision (e.g. confidence/credible interval) and measures of statistical heterogeneity. If comparing groups, describe the direction of the effect. | Not applicable |
|  | 20c | Present results of all investigations of possible causes of heterogeneity among study results. | Not applicable |
|  | 20d | Present results of all sensitivity analyses conducted to assess the robustness of the synthesized results. | Not applicable |
| Reporting biases | 21 | Present assessments of risk of bias due to missing results (arising from reporting biases) for each synthesis assessed. | Not applicable |
| Certainty of evidence | 22 | Present assessments of certainty (or confidence) in the body of evidence for each outcome assessed. | Not applicable |
| **DISCUSSION** | | |  |
| Discussion | 23a | Provide a general interpretation of the results in the context of other evidence. | 15-18 |
|  | 23b | Discuss any limitations of the evidence included in the review. | 18-19 |
|  | 23c | Discuss any limitations of the review processes used. | 18-19 |
|  | 23d | Discuss implications of the results for practice, policy, and future research. | 17-19 |
| **OTHER INFORMATION** | | |  |
| Registration and protocol | 24a | Provide registration information for the review, including register name and registration number, or state that the review was not registered. | 1 |
|  | 24b | Indicate where the review protocol can be accessed, or state that a protocol was not prepared. | 1,6 |
|  | 24c | Describe and explain any amendments to information provided at registration or in the protocol. | Not applicable |
| Support | 25 | Describe sources of financial or non-financial support for the review, and the role of the funders or sponsors in the review. | 20 |
| Competing interests | 26 | Declare any competing interests of review authors. | 20 |
| Availability of data, code and other materials | 27 | Report which of the following are publicly available and where they can be found: template data collection forms; data extracted from included studies; data used for all analyses; analytic code; any other materials used in the review. | Not applicable |

*From:*  Page MJ, McKenzie JE, Bossuyt PM, Boutron I, Hoffmann TC, Mulrow CD, et al. The PRISMA 2020 statement: an updated guideline for reporting systematic reviews. BMJ 2021;372:n71. doi: 10.1136/bmj.n71

For more information, visit: <http://www.prisma-statement.org/>

**Supplementary Table S3:** Systematic search strategy

Systematic searches were conducted in 6 databases including the Cochrane library, Medline, SocIndex, Cumulative Index to Nursing and Allied Health Literature (CINAHL), Scopus, and PsychInfo The search strategies for all databases are detailed below:

1. **SocIndex**

| **#** | **Query** |
| --- | --- |
| S29 | S24 AND S25 AND S27 |
| S28 | S24 AND S25 AND S26 |
| S27 | S20 OR S21 OR S22 OR S23 |
| S26 | S17 OR S18 OR S19 |
| S25 | S1 OR S2 OR S3 OR S4 OR S5 OR S6 OR S7 OR S8 OR S9 OR S10 OR S11 OR S12 OR S13 OR S14 OR S15 OR S16 |
| S24 | TI ( "intervention stud*" or "longitudinal stud*" or quasiexperimental or "quasi experimental" or quasi-experimental or "pseudo experimental" or "randomi?ed controlled trial" or "nonrandomi?ed trial" ) OR AB ( "intervention stud*" or "longitudinal stud*" or quasiexperimental or "quasi experimental" or quasi-experimental or "pseudo experimental" or "randomi?ed controlled trial" or "nonrandomi?ed trial" ) |
| S23 | TI ( unemployed or unemployment or "job loss" or jobseek* ) OR AB ( unemployed or unemployment or "job loss" or jobseek* ) |
| S22 | TI ( unemploy* or jobless or ((out or lost) N1 (work* or job* or employ*) ) OR AB ( unemploy* or jobless or ((out or lost) N1 (work* or job* or employ*) ) |
| S21 | DE "UNEMPLOYMENT & health" |
| S20 | DE "UNEMPLOYMENT" |
| S19 | DE "BODY weight" |
| S18 | TI ( obes* or obesity or obese or overweight or BMI or "body mass index" or "weight loss" or "weight gain" ) OR AB ( obes* or obesity or obese or overweight or BMI or "body mass index" or "weight loss" or "weight gain" ) |
| S17 | DE "OBESITY" |
| S16 | TI ( gardening or garden* or horticulture or "community garden" or "community garden*" or "organic agriculture" ) OR AB ( gardening or garden* or horticulture or "community garden" or "community garden*" or "organic agriculture" ) |
| S15 | TI ( "community participation" or "community engagement" or "community involvement" or community-based ) OR AB ( "community participation" or "community engagement" or "community involvement" or community-based ) |
| S14 | TI ( "cook* class*" or "nutrition class*" or "cook* lesson*" or "cooking intervention*" or "culinary intervention*" ) OR AB ( "cook* class*" or "nutrition class*" or "cook* lesson*" or "cooking intervention*" or "culinary intervention*" ) |
| S13 | TI ( "physical activity" N1 (intervention* or strateg* or program* or modif*) ) OR AB ( "physical activity" N1 (intervention* or strateg* or program* or modif*) ) |
| S12 | TI ( "employment program*" or "employment promotion program*" or reemployment or re-employment or "vocational intervention*" or "lifestyle program*" ) OR AB ( "employment program*" or "employment promotion program*" or reemployment or re-employment or "vocational intervention*" or "lifestyle program*" ) |
| S11 | TI ( diet* N2 (modif* or intervention* or strateg*) ) OR AB ( diet* N2 (modif* or intervention* or strateg*) ) |
| S10 | TI ( (lifestyle or "life style" N3 (chang* or intervention*) ) OR AB ( (lifestyle or "life style" N3 (chang* or intervention*) ) |
| S9 | TI ( obesity N2 (prevent* or intervention or strateg*) ) OR AB ( obesity N2 (prevent* or intervention or strateg*) ) |
| S8 | TI ( "vocational train*" or "return to work" or "career guidance" or "sheltered workshop*" ) OR AB ( "vocational train*" or "return to work" or "career guidance" or "sheltered workshop*" ) |
| S7 | TI ( "healthy eating" or "healthy diet" or "healthy nutrition" or fruit* or vegetable* or nutrition or "nutrition education" ) OR AB ( "healthy eating" or "healthy diet" or "healthy nutrition" or fruit* or vegetable* or nutrition or "nutrition education" ) |
| S6 | TI ( "physical activity" or exercise or fitness or "physical exercise" or sport or walk* or gym ) OR AB ( "physical activity" or exercise or fitness or "physical exercise" or sport or walk* or gym ) |
| S5 | DE "COMMUNITY life -- Social aspects" |
| S4 | DE "HEALTH promotion" |
| S3 | DE "EMPLOYMENT" |
| S2 | DE "FOOD consumption" |
| S1 | DE "EXERCISE" |

1. **PsychInfo**

| **#** | **Query** |
| --- | --- |
| S29 | S11 AND S23 AND S24 |
| S28 | S23 AND S24 AND S26 |
| S27 | S23 AND S24 AND S25 |
| S26 | S6 OR S10 |
| S25 | S5 OR S7 OR S9 |
| S24 | S1 OR S2 OR S3 OR S4 OR S8 OR S12 OR S13 OR S14 OR S15 OR S16 OR S17 OR S18 OR S19 OR S20 OR S21 OR S22 |
| S23 | TI ( "intervention stud*" or "longitudinal stud*" or quasiexperimental or "quasi experimental" or quasi-experimental or "pseudo experimental" or "randomised controlled trial" or "nonrandomised trial" or "qualitative stud*" ) OR AB ( "intervention stud*" or "longitudinal stud*" or quasiexperimental or "quasi experimental" or quasi-experimental or "pseudo experimental" or "randomised controlled trial" or "nonrandomised trial" or "qualitative stud*" ) |
| S22 | TI ( obes* N2 (prevention* or intervention* or strateg* or programm* ) OR AB ( obes* N2 (prevention* or intervention* or strateg* or programm* ) |
| S21 | TI ( diet* N2 (modif* or intervention* or strateg* ) OR AB ( diet* N2 (modif* or intervention* or strateg* ) |
| S20 | TI ( lifestyle or "life style" N3 (chang* or intervention* or modif* ) OR AB ( lifestyle or "life style" N3 (chang* or intervention* or modif* ) |
| S19 | TI ( "employment program*" or "employment promotion program*" or reemployment or re-employment or "vocational intervention*" or "lifestyle program*" ) OR AB ( "employment program*" or "employment promotion program*" or reemployment or re-employment or "vocational intervention*" or "lifestyle program*" ) |
| S18 | TI ( "physical activity" N1 (intervention* or strateg* or program* or modif* ) OR AB ( "physical activity" N1 (intervention* or strateg* or program* or modif* ) |
| S17 | TI ( gardening or garden* or horticulture or "community garden" or "community garden*" or "organic agriculture" ) OR AB ( gardening or garden* or horticulture or "community garden" or "community garden*" or "organic agriculture" ) |
| S16 | TI ( "cook* class*" or "nutrition class*" or "cook* lesson*" or "cooking intervention*" or "culinary intervention*" ) OR AB ( "cook* class*" or "nutrition class*" or "cook* lesson*" or "cooking intervention*" or "culinary intervention*" ) |
| S15 | TI ( "vocational train*" or "return to work" or "career guidance" or "sheltered workshop*" ) OR AB ( "vocational train*" or "return to work" or "career guidance" or "sheltered workshop*" ) |
| S14 | TI ( "healthy eating" or "healthy diet*" or "healthy nutrition" or fruit* or vegetable* or nutrition or "nutrition education" ) OR AB ( "healthy eating" or "healthy diet*" or "healthy nutrition" or fruit* or vegetable* or nutrition or "nutrition education" ) |
| S13 | TI ( "physical activity" or exercise or fitness or "physical exercise" or sport or walk* or gym ) OR AB ( "physical activity" or exercise or fitness or "physical exercise" or sport or walk* or gym ) |
| S12 | TI "Weight Reduction Program*" OR AB "Weight Reduction Program*" |
| S11 | TI ( "vulnerable population*" or "vulnerable group*" or "deprived communit*" or "disadvantaged group*" or "socially disadvantaged group*" or "low income population*" ) OR AB ( "vulnerable population*" or "vulnerable group*" or "deprived communit*" or "disadvantaged group*" or "socially disadvantaged group*" or "low income population*" ) |
| S10 | TI ( unemployed or unemployment or "job loss" or jobseek* ) OR AB ( unemployed or unemployment or "job loss" or jobseek* ) |
| S9 | TI ( obes* or obesity or obese or overweight or BMI or "body mass index" or "weight loss" or "weight gain" ) OR AB ( obes* or obesity or obese or overweight or BMI or "body mass index" or "weight loss" or "weight gain" ) |
| S8 | DE "Health Promotion" |
| S7 | DE "Body Mass Index" OR DE "Body Size" OR DE "Body Weight" |
| S6 | DE "Unemployment" |
| S5 | DE "Obesity" |
| S4 | DE "Nutrition" OR DE "Alcoholic Beverages" OR DE "Beverages (Nonalcoholic)" OR DE "Calories" OR DE "Carbohydrates" OR DE "Dietary Supplements" OR DE "Diets" OR DE "Energy Drink" OR DE "Food" OR DE "Mealtimes" |
| S3 | DE "Horticulture Therapy" |
| S2 | DE "Exercise" OR DE "Aerobic Exercise" OR DE "Weightlifting" OR DE "Yoga" |
| S1 | DE "Physical Activity" OR DE "Actigraphy" OR DE "Exercise" |

1. **Medline Complete**

| **#** | **Query** |
| --- | --- |
| S40 | S19 AND S37 |
| S39 | S19 AND S36 |
| S38 | S19 AND S35 |
| S37 | S31 AND S34 |
| S36 | S31 AND S33 |
| S35 | S31 AND S32 |
| S34 | S13 OR S27 |
| S33 | S5 OR S11 OR S12 OR S26 |
| S32 | S22 OR S29 OR S14 OR S30 |
| S31 | S1 OR S11 OR S25 OR S26 OR S27 OR S10 OR S15 OR S16 OR S17 OR S18 OR S20 OR S21 OR S22 OR S23 OR S24 OR S25 OR S28 OR S29 |
| S30 | TI ( unemployed or unemployment or "job loss" or jobseek* ) OR AB ( unemployed or unemployment or "job loss" or jobseek* ) |
| S29 | TI ( gardening or garden* or horticulture or "community garden" or "community garden*" or "organic agriculture" ) OR AB ( gardening or garden or horticulture or "community garden" or "community garden*" or "organic agriculture" ) |
| S28 | TI ( "community participation" or "community engagement" or "community involvement" or community-based) OR AB ( "community participation" or "community engagement" or "community involvement" or community-based) |
| S27 | TI ( "vulnerable population*" or "vulnerable group*" or "deprived communit*" or "disadvantaged group*" or "socially disadvantaged group*" or "low income population*") OR AB ( "vulnerable populations" or "vulnerable group*" or "deprived communit*" or "disadvantaged group*" or "socially disadvantaged group*" or "low income population*") |
| S26 | (MH "Overweight+") OR (MH "Body Mass Index") |
| S25 | (MH "Community Participation") OR "community intervention" OR (MH "Community Networks") |
| S24 | TI ( "cook* class*" or "nutrition class*" or "cook* lesson*" or "cooking intervention*" or "culinary intervention*") OR AB ( "cook* class*" or "nutrition class*" or "cook* lesson*" or "cooking intervention*" or "culinary intervention*") |
| S23 | (MH "Weight Reduction Programs+") |
| S22 | (MH "Vegetables") OR (MH "Fruit and Vegetable Juices") |
| S21 | TI ( "physical activity" N1 (intervention* or strateg* or program* or modif*) ) OR AB ( "physical activity" N1 (intervention* or strateg* or program* or modif*) ) |
| S20 | TI ( "employment program*" or "employment promotion program*" or reemployment or re-employment or "vocational intervention*" or "lifestyle program*" ) OR AB ( "employment program*" or "employment promotion program*" or reemployment or re-employment or "vocational intervention*" or "lifestyle program*" ) |
| S19 | TI ("intervention stud*" or "longitudinal stud*" or quasiexperimental or "quasi experimental" or quasi-experimental or "pseudo experimental" ) OR AB ( "intervention stud*" or "longitudinal stud*" or quasiexperimental or "quasi experimental" or quasi-experimental or "pseudo experimental" ) |
| S18 | TI ( diet* N2 (modif* or intervention* or strateg*) ) OR AB ( diet* N2 (modif* or intervention* or strateg*) ) |
| S17 | TI (lifestyle or "life style" N3 (chang* or intervention*) ) OR AB (lifestyle or "life style" N2 (chang* or intervention*) ) |
| S16 | TI ( obesity N2 (prevent* or intervention or strateg*) ) OR AB ( obesity N2 (prevent* or intervention or strateg*) ) |
| S15 | (MH "Health Promotion+") |
| S14 | TI ( unemploy* or jobless or ((out or lost) N1(work* or job* or employ*) ) OR AB ( unemploy* or jobless or ((out or lost) N1(work* or job* or employ*) ) |
| S13 | (MH "Vulnerable Populations") |
| S12 | (MH "Weight Loss+") OR (MH "Body Weight Maintenance") OR (MH "Weight Gain") OR (MH "Body Weight+") |
| S11 | (MH "Obesity+") |
| S10 | TI ("vocational train*" or "return to work" or "career guidance" or "sheltered workshop*") OR AB ( "vocational train*" or "return to work" or "career guidance" or "sheltered workshop*") |
| S9 | unemploy* or jobless or ((out or lost) N1 (work* or job* or employ*) |
| S8 | (MH "Employment, Supported") OR (MH "Employment+") OR (MH "Job Application") |
| S7 | TI ( "healthy eating" or "healthy diet" or "healthy nutrition" or fruit* or vegetable* or nutrition or "nutrition education") OR AB ( "healthy eating" or "healthy diet" or "healthy nutrition" or fruit* or vegetable* or nutrition or "nutrition education") |
| S6 | TI ( "physical activity" or exercise or fitness or "physical exercise" or sport or walk* or gym ) OR AB ( "physical activity" or exercise or fitness or "physical exercise" or sport or walk* or gym ) |
| S5 | TI ( obes* or obesity or obese or overweight or BMI or "body mass index" or "weight loss" or "weight gain") OR AB ( obes* or obesity or obese or overweight or BMI or "body mass index" or "weight loss" or "weight gain") |
| S4 | (MH "Diet+") OR (MH "Diet, Healthy") |
| S3 | (MH "Unemployment") |
| S2 | (MH "Gardening") OR (MH "Organic Agriculture") |
| S1 | (MH "Exercise+") |

1. **CINAHL Complete**

| **#** | **Query** |
| --- | --- |
| S33 | S26 AND S29 AND S30 |
| S32 | S26 AND S28 AND S30 |
| S31 | S26 AND S27 AND S30 |
| S30 | TI ( "intervention stud*" or "longitudinal stud*" or quasiexperimental or "quasi experimental" or quasi-experimental or "pseudo experimental" or "randomised controlled trial" or "nonrandomised trial" ) OR AB ( "intervention stud*" or "longitudinal stud*" or quasiexperimental or "quasi experimental" or quasi-experimental or "pseudo experimental" or "randomised controlled trial" or "nonrandomised trial" ) |
| S29 | S9 OR S15 |
| S28 | S7 OR S14 |
| S27 | S6 OR S8 OR S13 |
| S26 | S1 OR S2 OR S3 OR S4 OR S5 OR S10 OR S11 OR S12 OR S16 OR S17 OR S18 OR S19 OR S20 OR S21 OR S22 OR S23 OR S24 OR S25 |
| S25 | TI ( diet* N2 (modif* or intervention* or strateg*) ) OR AB ( diet* N2 (modif* or intervention* or strateg*) ) |
| S24 | TI ( lifestyle or "life style" N3 (chang* or intervention* or modif* ) OR AB ( lifestyle or "life style" N3 (chang* or intervention* or modif* ) |
| S23 | TI ( "employment program*" or "employment promotion program*" or reemployment or re-employment or "vocational intervention*" or "lifestyle program*" ) OR AB ( "employment program*" or "employment promotion program*" or reemployment or re-employment or "vocational intervention*" or "lifestyle program*" ) |
| S22 | TI ( "physical activity" N1 (intervention* or strateg* or program* or modif* ) OR AB ( "physical activity" N1 (intervention* or strateg* or program* or modif* ) |
| S21 | TI ( gardening or garden* or horticulture or "community garden" or "community garden*" or "organic agriculture" ) OR AB ( gardening or garden* or horticulture or "community garden" or "community garden*" or "organic agriculture" ) |
| S20 | TI ( "cook* class*" or "nutrition class*" or "cook* lesson*" or "cooking intervention*" or "culinary intervention*" ) OR AB ( "cook* class*" or "nutrition class*" or "cook* lesson*" or "cooking intervention*" or "culinary intervention*" ) |
| S19 | TI ( "vocational train*" or "return to work" or "career guidance" or "sheltered workshop*" ) OR AB ( "vocational train*" or "return to work" or "career guidance" or "sheltered workshop*" ) |
| S18 | TI ( "healthy eating" or "healthy diet*" or "healthy nutrition" or fruit* or vegetable* or nutrition or "nutrition education" ) OR AB ( "healthy eating" or "healthy diet*" or "healthy nutrition" or fruit* or vegetable* or nutrition or "nutrition education" ) |
| S17 | TI ( "physical activity" or exercise or fitness or "physical exercise" or sport or walk* or gym ) OR AB ( "physical activity" or exercise or fitness or "physical exercise" or sport or walk* or gym ) |
| S16 | (MH "Weight Reduction Programs") |
| S15 | TI ( "vulnerable population*" or "vulnerable group*" or "deprived communit*" or "disadvantaged group*" or "socially disadvantaged group*" or "low income population*" ) OR AB ( "vulnerable population*" or "vulnerable group*" or "deprived communit*" or "disadvantaged group*" or "socially disadvantaged group*" or "low income population*" ) |
| S14 | TI ( unemployed or unemployment or "job loss" or jobseek* ) OR AB ( unemployed or unemployment or "job loss" or jobseek* ) |
| S13 | TI ( obes* or obesity or obese or overweight or BMI or "body mass index" or "weight loss" or "weight gain" ) OR AB ( obes* or obesity or obese or overweight or BMI or "body mass index" or "weight loss" or "weight gain" ) |
| S12 | (MH "Vegetables+") |
| S11 | (MH "Fruit+") |
| S10 | (MH "Health Promotion") |
| S9 | (MH "Special Populations") |
| S8 | (MH "Body Mass Index") |
| S7 | (MH "Unemployment") |
| S6 | (MH "Obesity+") |
| S5 | (MH "Nutrition+") |
| S4 | (MH "Diet+") |
| S3 | (MH "Horticulture") |
| S2 | (MH "Exercise+") |
| S1 | (MH "Physical Activity") |

1. **Cochrane Library**

ID Search

#1 MeSH descriptor: [Exercise] explode all trees

#2 MeSH descriptor: [Organic Agriculture] explode all trees

#3 MeSH descriptor: [Diet] explode all trees

#4 MeSH descriptor: [Health Promotion] explode all trees

#5 MeSH descriptor: [Diet, Healthy] explode all trees

#6 MeSH descriptor: [Fruit] 2 tree(s) exploded

#7 ("physical activity" or exercise or fitness or "physical exercise" or sport or walk* or gym):ti,ab,kw

#8 ("healthy eating" or "healthy diet" or "healthy nutrition" or fruit* or vegetable* or nutrition or "nutrition education"):ti,ab,kw

#9 ("vocational train*" or "return to work" or "career guidance" or "sheltered workshop*"):ti,ab,kw

#10 (obesity Near/2 (prevent* or intervention or strateg*)):ti,ab,kw

#11 ((lifestyle or "life style" Near/3 (chang* or intervention*) )):ti,ab,kw

#12 (( diet* Near/2 (modif* or intervention* or strateg*) )):ti,ab,kw

#13 ("physical activity" Near/1 (intervention* or strateg* or program* or modif*)):ti,ab,kw

#14 ("employment program*" or "employment promotion program*" or reemployment or re-employment or "vocational intervention*" or "lifestyle program*"):ti,ab,kw

#15 ("cook* class*" or "nutrition class*" or "cook* lesson*" or "cooking intervention*" or "culinary intervention*"):ti,ab,kw

#16 ("community participation" or "community engagement" or "community involvement" or community-based):ti,ab,kw

#17 (gardening or garden* or horticulture or "community garden" or "community garden*" or "organic agriculture"):ti,ab,kw

#18 MeSH descriptor: [Obesity] 4 tree(s) exploded

#19 (obes* or obesity or obese or overweight or BMI or "body mass index" or "weight loss" or "weight gain"):ti,ab,kw

#20 MeSH descriptor: [Unemployment] explode all trees

#21 (unemployed or unemployment or "job loss" or jobseek*):ti,ab,kw

#22 MeSH descriptor: [Vulnerable Populations] 1 tree(s) exploded

#23 ("vulnerable population*" or "vulnerable group*" or "deprived communit*" or "disadvantaged group*" or "socially disadvantaged group*" or "low income population*"):ti,ab,kw

#24 ("intervention stud*" or "longitudinal stud*" or quasiexperimental or "quasi experimental" or quasi-experimental or "pseudo experimental" or "randomi?ed controlled trial" or "nonrandomi?ed trial"):ti,ab,kw

#25 #1 or #2 or #3 or #4 or #5 or #6 or #7 or #8 or #9 or #10 or #11 or #12 or #13 or #14 or #15 or #16 or #17

#26 #18 or #19

#27 #20 or #21

#28 #22 or #23

#29 #25 and #26 and #24

#30 #25 and #27 and #24

#31 #25 and #28 and #24

1. **SCOPUS**

| ( unemployment OR unemployed OR unemployment OR "job loss" OR jobseek* OR "job seek*" OR unemploy OR jobless ) AND ( ( TITLE-ABS-KEY ( exercise OR "physical activity" OR fitness OR "physical exercise" OR sport OR walk* OR gym OR "physical activity" W/2 ( intervention OR strategy OR program OR modif* ) ) ) OR ( TITLE-ABS-KEY ( diet* OR "healthy eating" OR "healthy diet" OR "healthy nutrition" OR fruit* OR vegetable* OR nutrition OR "nutrition education" OR diet* W/2 ( modif* OR intervention* OR strategy ) OR lifestyle OR "life style" W/3 ( chang* OR intervention* ) ) ) OR ( TITLE-ABS-KEY ( obesity W/2 ( prevent* OR intervention OR strateg* ) OR "health promotion" OR "weight reduction program*" ) ) OR ( gardening OR "organic agriculture" OR horticulture OR "community garden" OR "community garden*" OR "cook* class*" OR "nutrition class*" OR "cook* lesson*" OR "cooking intervention" OR "culinary intervention" ) OR ( "supported employment" OR "job application" OR "vocational train*" OR "return to work" OR "career guidance" OR "sheltered workshop*" OR "employment program*" ) OR ( "lifestyle program" OR "community participation" OR "community intervention" OR "community network*" OR "community participation" OR "community engagement" OR "community involvement" OR "community-based" ) AND ( obesity OR obese OR overweight OR bmi OR "body mass index" OR "weight loss" OR "weight gain" ) AND ( "intervention stud*" OR "longitudinal stud*" OR quasiexperimental OR "quasi experimental" OR quasi-experimental OR "pseudo experimental" OR "randomi?ed controlled trial" OR "nonrandomi?ed trial" ) ) |
| --- |
